# Grinding Beads Influence Microbial DNA Extraction from Organic-Rich Sub-Seafloor Sediment

**DOI:** 10.3390/microorganisms10122505

**Published:** 2022-12-18

**Authors:** Jingjing Niu, Hong Chen, Lanlan Cai, Maoqiu He, Rui Zhang, Long Wang

**Affiliations:** 1 State Key Laboratory of Marine Environmental Science, Fujian Key Laboratory of Marine Carbon Sequestration, College of Ocean and Earth Sciences, Xiamen University, Xiamen 361102, China; 2 Department of Ocean Science, The Hong Kong University of Science and Technology, Hong Kong, China; 3 Fishery College, Zhejiang Ocean University, Zhoushan 316022, China; 4 Institute for Advanced Study, Shenzhen University, Shenzhen 518061, China; 5 Southern Marine Science and Engineering Guangdong Laboratory (Zhuhai), Zhuhai 519080, China

**Keywords:** DNA extraction, grinding bead, sub-seafloor sediment, Baltic Sea

## Abstract

Sub-seafloor sediment is the largest microbial habitat on Earth. The study of microbes in sub-seafloor sediment is largely limited by the technical challenge of acquiring ambient microbial DNA because of sediment heterogeneity. Changes in the extraction method, even just by one step, can affect the extraction yields for complicated sediment samples. In this work, sub-seafloor sediment samples from the Baltic Sea with high organic carbon content were used to evaluate the influence of different grinding beads on DNA extraction. We found that the grinding beads can affect the DNA extraction from the organic-matter- and biosiliceous-clay-rich samples. A mixture of 0.5-mm and 0.1-mm grinding beads exhibited higher DNA yields and recovered more unique taxa than other bead combinations, such as *Stenotrophomonas* from Gammaproteobacteria and *Leptotrichia* from Fusobacteria; therefore, these beads are more suitable than the others for DNA extraction from the samples used in this study. This advantage might be magnified in samples with high biomass. On the contrary, the use of only small beads might lead to underestimation for certain Gram-positive strains. Overall, the discovery of abundant widespread deep biosphere clades in our samples indicated that our optimized DNA extraction method successfully recovered the in situ microbial community.

## 1. Introduction

The fundamental step for determining microbial diversity and function in complex environmental samples is extraction of high quantities of high-quality DNA; however, this remains challenging [1]. Except for the DNA yield, the modification of a single step in the DNA extraction protocol may influence the resulting microbial community diversity [1,2,3], primarily due to the structural difference of cell walls [4,5,6,7,8,9]. Previous studies have implied that the combination of different lytic strategies (bead beating, a cock tail of lytic enzymes, etc.) can obtain the best representation of microbial diversity [10]. For example, the physical disruption was deemed to improve the recovery of Actinobacteria, Nitrospirota, Chloroflexota, and Alphaproteobacteria [11]. It is not possible to simply apply one protocol to extract DNA from different environmental samples. The extraction of microbial DNA from sub-seafloor sediment is more challenging than that from water, soil, or marine surface sediment because of the diversity of sediment types (e.g., fine sand, silt, and clay), low biomass, complex structures (e.g., spores and cysts), and multiple potential enzyme inhibitors (e.g., heavy metals, colloids, fulvic acids, and humic acids) that exist in the deep biosphere [12]. Sediment microbial DNA can be extracted by separating the cells from the sediment particles before lysis (cell extraction method) or by direct lysis of cells in the sediment without separation (direct lysis method) [13].

The cell extraction method is time-consuming, has a low recovery rate, and may introduce extraction bias for different microbial groups [14]. By contrast, several studies have shown that the direct lysis method provides a high DNA yield, ribotype richness, and diversity and is a better method than cell extraction for investigating microbial diversity in sediments [15,16,17,18,19,20]. Direct lysis can be achieved by mechanical disruption (e.g., by freeze-thawing, bead-mill homogenization, and/or sonication), chemical lysis (e.g., with detergents, sodium chloride, and/or chaotropic agents), and enzymatic lysis (e.g., by lysozyme, proteinase K, achromopeptidase, and/or protease E) [12,21,22,23]. Moré et al. (1994) suggested that mechanical treatment is more efficient and less selective than chemical lysis [24]. Various investigations into soil microbial DNA extraction methods have found that bead milling produces higher DNA yields than other mechanical disruption methods [15,22,24,25,26,27]. In this method, the grinding beads effectively separate and fragment cells by interaction with the sediment matrix. Some studies have investigated the impact of the milling time and intensity on the DNA yield and recovery rate [28]. However, there have been few specific reports on the influence of various parameters of the grinding beads on DNA extraction [29].

For microbial DNA extraction from sediment samples, commercial bead-based DNA extraction kits are commonly used. In contrast to manual extraction of DNA [25], which is time-consuming, commercial DNA extraction kits are more suitable for molecular ecological investigations of large numbers of samples because they use prepared reagents and streamlined processes [17,25]. Over the past two decades, many commercial kits have been developed and used for extraction of DNA from various environmental samples (e.g., terrestrial soil, marine sediment, permafrost, and sludge) [11,17,21,26,30]. However, commercial DNA extraction kits designed for sediment samples are not widely available. In some studies, commercial soil DNA extraction kits were used to recover microbial DNA from marine sediment. Lever et al. (2015) found that the use of these kits resulted in lower DNA yields [31], and Morono et al. (2014) found that almost two-thirds of all microbial cells were not destroyed using commercial kits [3]. Therefore, to improve the suitability and effectiveness of these kits for application to microbial DNA extraction from sediment samples, it is essential to modify the parameters of the grinding beads in commercial soil kits to facilitate the isolation and disruption of microbial cells without causing excessive fragmentation of the DNA.

The Baltic Sea basin is one of the world’s largest intra-continental basins [32]. The Baltic Sea environment has undergone dramatic changes over time because of switching between freshwater with low organic carbon deposition periods and seawater with high organic carbon deposition periods [32]. These climate changes are reflected in the high-resolution sediments of the Baltic Sea [33]. The Baltic Sea is also characterized by rapid sedimentation rates and high levels of organic matter and nutrients that support the growth of benthic microbial communities [34]. Thus, the Baltic Sea serves as a natural laboratory for studying the effects of sediment age, energy availability, and changing salinity throughout time on microbial communities in the deep sub-seafloor. It also provides an excellent experimental material for the optimization of sediment microbial DNA extraction methods.

## 2. Materials and Methods

### 2.1. Sample Collection

**Figure 1:**
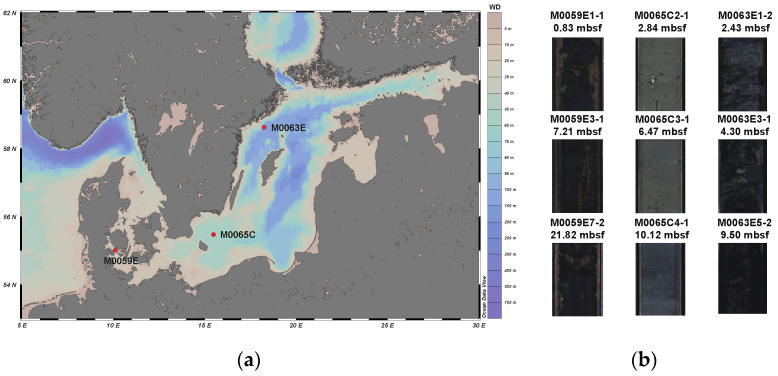
(**a**) Coring sites of the IODP drilling expedition 347 with the specific holes sampled. (**b**) The picture of the neighbor section of sediment samples used in this study. WD: water depth, mbsf: meters below seafloor.

The Baltic Sea is an organic-rich basin with a high sedimentation rate of 100–500 cm/ky [34]. During the IODP Expedition 347 in the Baltic Sea in 2013, sediments were sampled from three boreholes: M0059E, M0065C, and M0063E. M0059E was located in a shallow region (depth of 37.1 m) at the entrance to the Baltic Sea. In this area, marine water from the North Sea flows into the Baltic Sea [33]. M0065C was situated in the middle of the Baltic Sea and the depth was 84.3 m. M0063E was located in the deepest part (437.1 m) of the Baltic Sea basin. This area of the sea experiences periodic anoxia [35] (Figure 1a). All samples were packaged in Whirl-Pak sampling bags on board, transported to the laboratory on dry ice, and stored at −80 °C until required for analysis. Three samples from different depths at each borehole (a total of nine samples) were used for DNA extraction (Figure 1b).

For the different boreholes, there were some variations in the physical properties (Table S1 and Figure S1). The major lithology of the M0059E samples used in this study was biosiliceous clay, containing 68–89% clay minerals and 5–27% biogenic silica (primarily diatoms, silicoflagellates, and sponge spicules) [34]. The samples at borehole M0065C were dark greenish gray organic-rich clay with very low silt and sand contents [34]. The sediments from M0063E were organic-rich diatom-bearing black clays, which had very low contents of silt and sand [34]. For samples from all three boreholes, the water content (wet mass) and fractional porosity decreased with depth, with the M0059E samples having higher water contents and porosities than the samples from the other two boreholes. Compared with the samples from M0059E and M0063E, the M0065C samples had higher particle densities.

In terms of chemical parameters, vertical and horizontal variations were evident (Table S1 and Figure S1). The majority of these values gradually decreased as the distance of the sampling borehole increased from the entrance to the Baltic Sea (M0059E) to the inner Baltic Sea (M0065C and M0063E). The salinity clearly decreased with depth, ranging from 26.69 to 32.15 (average: 30.31 ± 2.90) at M0059E, 13.90 to 16.40 (average: 15.37 ± 1.31) at M0065C, and 11.80 to 13.00 (average: 13.10 ± 1.35) at M0063E. The total organic matter (TOC) content, and ammonium (NH_4_^+^) and phosphate (PO_4_^3−^) concentrations were positively correlated with the salinity (Spearman correlations of *p* = 0.036, *p* = 0.001, and *p* = 0.0016, respectively). Although scattering of the data was observed in some parts of the profiles, the sulfate (SO_4_^2−^) concentrations in all sediment samples in this work were generally <0.15 mM. The sulfate concentrations varied from 0.030 to 0.070 mM (average: 0.043 ± 0.023 mM) at M0059E, 0.010 to 0.100 mM (average: 0.046 ± 0.047 mM) at M0065C, and 0.050 to 0.120 mM (average: 0.070 ± 0.044 mM) at M0063E.

### 2.2. Combinations of Grinding Beads

**Figure 2:**
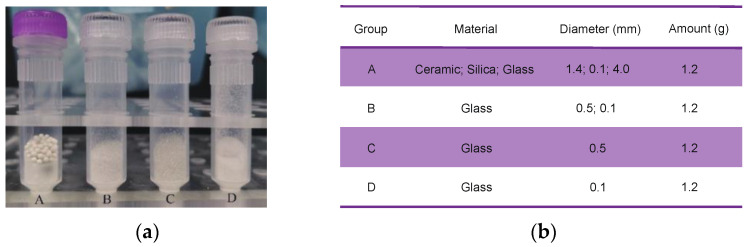
(**a**) Four combinations of grinding beads. (**b**) Specific parameters for each combination of grinding beads.

Four different types of beads made from various materials and with different diameters were used in different amounts. The results were used to compare the efficiency of different bead combinations on environmental microbial DNA extraction from sediment samples (Figure 2). Group A contained 1.2 g of 1.4-mm ceramic beads, 0.1-mm silica beads, and a 4-mm glass bead (from the FastDNA^®^ SPIN Kit for soil, MP Biomedicals, Santa Ana, CA, USA); group B contained 0.6 g of 0.1-mm glass beads and 0.6 g of 0.5-mm glass beads; group C contained 1.2 g of 0.5-mm glass beads; and group D contained 1.2 g of 0.1-mm glass beads. Three parallel sets of sediment samples were lysed using each group of beads. The results for the experiments were labeled according to the sediment sample location and extraction method, for example, M0059E1-1A, M0059E1-1B, M0059E1-1C, and M0059E1-1D for sediment sample M0059E1-1 extracted using the group A, B, C, and D beads, respectively.

### 2.3. DNA Extraction

Three DNA extractions using the grinding beads in each group were conducted using the FastDNA^®^ SPIN Kit for soil (MP Biomedicals) following the manufacturer’s instructions with some modifications. The following extraction steps were used (Figure S2):

(I) Each sediment sample was thawed at 4 °C until it was soft enough to scrape off the surface with a sterile knife. Then, 0.5 g of the wet sediment sample was added to a 2-mL lysis tube with one of the groups of beads.
(II) Lysis buffer (1 mL) containing 978 μL of sodium phosphate buffer and 122 μL of MT buffer was added to the lysis tube. The tube was then secured horizontally on a Vortex-Genie Adapter (Thermo Fisher Scientific, Waltham, MA, USA) and vortex mixed gently to dissolve the sediment and mix it with the beads and buffer.
(III) The lysis tube was homogenized in a FastPrep instrument twice for 45 s at 6.0 m s^−1^ with a 5 min interval in an ice bath between the two cycles. This step was used to disrupt the cell wall and release nucleic acids.
(IV) The sediment/bead mixture was centrifuged at 14,000× *g* for 10 min at 4 °C to pelletize any debris such as insoluble cellular debris and the lysis matrix.
(V) The supernatant was transferred to a clean 2-mL microcentrifuge tube. PPS (250 μL) was added to separate the solubilized nucleic acids from the cellular debris and lysis matrix. After inverting the tube 10 times, it was centrifuged at 14,000× *g* for 5 min (4 °C).
(VI) The supernatant was transferred to two clean 2-mL microcentrifuge tubes (approximately 0.5 mL each) and then resuspended with 0.5 mL of binding matrix. After vortex mixing, the tubes were placed in an ice bath for 15 min.
(VII) For each of two tubes, 300 μL of the supernatant was carefully removed and discarded to avoid disturbing the settled binding matrix. The binding matrix in the remaining amount of supernatant was gently resuspended. The remaining 700 μL of the mixtures was transferred to a spin filter and centrifuged at 14,000× *g* for 2 min (4 °C). The spin filter was then emptied and reused for another tube of the mixture.
(VIII) Prepared SEWS-M (500 μL with an appropriate volume of ethanol added) was added to solubilize any impurities. The pellet was gently resuspended using liquid from a pipet tip, which was followed by centrifugation at 14,000× *g* for 1 min (4 °C). The tube was then emptied. After washing the spin filter three times, it was centrifuged at 14,000× *g* for 2 min without any added liquid to completely remove SEWS-M.
(IX) The catch tube was replaced with a new, clean catch tube, and the spin filter was air dried for 10 min at room temperature to remove the residual alcohol.
(X) The binding matrix (above the spin filter) was gently resuspended in 100 μL of DES and incubated for 5 min at 55 °C in a heat block or water bath.
(XI) The tube was centrifuged at 14,000× *g* for 1 min to bring the eluted DNA into the clean catch tube and the spin filter was discarded. The DNA samples were stored at −20 °C until required for amplification.

The DNA concentrations were determined using the Quant-iT dsDNA BR assay kit and a Qubit fluorometer (Invitrogen GmbH, Karlsruhe, Germany). DNA purity was measured with a spectrophotometer at 260 and 280 nm (*A_260_* and *A_280_*) using a NanoDrop ND-2000 (Thermo Fisher Scientific, Waltham, MA, USA). Nucleic acids were measured using the peak at 260 nm, and proteins and phenolics were measured using the peak at 280 nm. Pure DNA preparations have an expected *A_260_*/*A_280_* ratio of 1.8.

### 2.4. Analyses of Sequences and Statistics

The DNA from the three parallel extractions for each group was mixed for the following experiments. The primer pair 515F (5′-GTG YCA GCM GCC GCG GTA A-3′) [36] and 806R (5′-GGA CTA CNV GGG TWT CTA AT-3′) [37] was used to amplify the V4 region of the bacterial and archaeal 16S rRNA gene. Three parallel samples of the PCR products were mixed and purified with magnetic beads. The DNA concentration was detected by a microplate reader, and the fragment size was detected by agarose gel electrophoresis. The library was quantified to 10 nM, and PE250 paired-end sequencing was performed according to the Illumina MiSeq (Illumina, San Diego, CA, USA) instrument manual by GENEWIZ (Suzhou, China). Sequencing data were analyzed and visualized using QIIME 2 (Version 2020.11.1) [38]. Demultiplexed reads were quality filtered using the QIIME2 plugin quality filter with the default settings [39]. Reads were then denoised, chimera checked, and dereplicated using a DADA2 denoise-paired plugin [40]. Amplicon sequence variants (ASVs) were classified based on the 100% non-clustered unique sequences obtained by DADA2 [40]. Using the qiime feature-table rarefy, the outputs of DADA2 were rarefied to the lowest sample at 5631 sequences. Past (Version 4.02) was used to calculate the diversity indices, including Chao-1 and Shannon indices [41]. Taxonomic assignments were performed by qiime feature-classifier classify-sklearn using a pre-trained Naïve−Bayes classifier with the SILVA database (Version 138) [42]. Taxa bar plots were built using the plugin qiime taxa bar plot with the feature table.

Statistical analyses relating to DNA purity, DNA concentration, and diversities were performed using one-way analysis of variance (ANOVA) by Tukey’s test. Significant results were subjected to pairwise comparisons using the Student’s *t*-test. All statistical analyses and data visualization were performed using GraphPad Prism (Version 9.3.1, Dotmatics, San Diego, CA, USA).

## 3. Results

### 3.1. Sample Description

We compared the efficiencies of four different groups of grinding beads for DNA extraction from Baltic Sea sub-seafloor sediment samples with a wide range of physicochemical and microbial characteristics (Table S1 and Figure S1). The cell abundances of the nine samples (three samples collected at each of three sites) varied by nearly 30-fold, with a range of 2.67 × 10^8^ cells/cm^3^ in M0065C4-1 to 7.57 × 10^9^ cells/cm^3^ in M0063E3-1 [43]. All of the sediment samples were fine clay minerals with a clay content of up to 89% and had low water contents that decreased with sediment depth at each sampling site. The trend for the NH_4_^+^ concentration at M0059E and M0063E was the reverse of that for water, and the concentration peaked in M0059E7-2 at 36.99 mM. The salinity varied widely among the sampling sites, with M0059E being classed as a marine sediment and M0063E and M0065C as brackish sediments. All nine samples had high TOC contents (1.85–7.40%), and oxygen and sulfate were depleted.

### 3.2. Effect of Grinding Beads on DNA Quality

**Figure 3:**
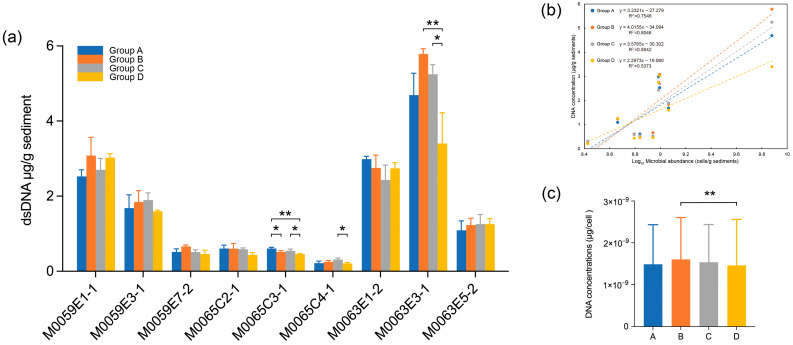
(**a**) For the same sediment sample, the differences in DNA concentrations extracted by four grinding bead combinations. (**b**) Relationship between DNA concentration and microbial abundance (log_10_ transformed), *p* < 0.05 by linear regression. (**c**) The differences in normalized DNA concentrations extracted by four grinding bead combinations. The line in data plots indicate median value with standard deviation (SD). Asterisks denote significance, “*” represent *p* < 0.05, “**” represent *p* < 0.01, respectively.

Concentration is an important parameter when evaluating the efficiency of DNA extraction. In the present study, significant differences were observed among the four groups of grinding beads used for extraction of DNA from M0065C3-1, M0065C4-1, and M0063E3-1. The grinding beads in groups A, C, and B performed the best (one-way ANOVA, *p* < 0.05; Figure 3a). The group D beads produced the lowest quantity of double-stranded DNA among the four groups of beads (Student’s *t*-test, *p* < 0.05). Significant positive correlations between DNA concentration and cell abundance were found for all four groups of beads (Spearman, *p* < 0.05; Figure 3b). The grinding beads in groups B and C showed the best fit with the cell abundance and the highest average DNA concentration, which was consistent with their good performance for DNA extraction compared with group D. After controlling for the influence of cell abundance, the DNA concentrations showed no significant relationships with the other tested parameters. However, normalized DNA concentration was significantly higher in group B than in group D (Student’s *t*-test, *p* = 0.0085; Figure 3c).

The *A_260_*/*A_280_* ratio was measured using Nanodrop and used to evaluate the purity of the DNA extracted in this work. This ratio ranged from 1.40 to 1.75 (1.535 ± 0.074) for all DNA, which indicated high purity and a low level of protein contamination. Although different grinding beads did not influence the purity of the DNA, significant variation was found among the nine samples (one-way ANOVA, *p* < 10^−4^; Figure S3a). The DNA purity was higher at M0063E, which had low a TOC content (1.560 ± 0.080), than at M0059E (1.510 ± 0.084) or M0065C (1.534 ± 0.090), which had high TOC contents. The purities of the DNA extracted using all four groups of grinding beads were significantly positively correlated with the sulfate concentration (Spearman, *p* < 0.05; Figure S3b).

### 3.3. Effect of Grinding Beads on the Microbial Community Alpha Diversity

By amplifying and sequencing the V4 region 16S rRNA gene, >2.5 million raw sequences were obtained. After quality control, at least 38,260 reads with an average sequence length of 293 bp were left in each sample for further analyses (Table S2). The DADA2 algorithm was used to dereplicate the high-quality sequences, and a total of 10,031 ASVs were obtained. For the DADA2 community, groups C and D had significantly higher Chao-1 indices than group A at M0063E (Student’s *t*-test, *p* < 0.05; Table S3 and Figure S4). Overall, the DADA2 algorithm could identify high community diversity and was sensitive to the differences caused by the grinding beads in the DNA extraction process. Consequently, all further analyses were based on the ASV community of DADA2. We also found consistent relationships between community diversity and environmental parameters. The Shannon index in groups B and D was negatively correlated in depth, but only group B was positively correlated with water content (Spearman, *p* < 0.05).

### 3.4. Effect of Grinding Beads on the Microbial Community Composition

**Figure 4:**
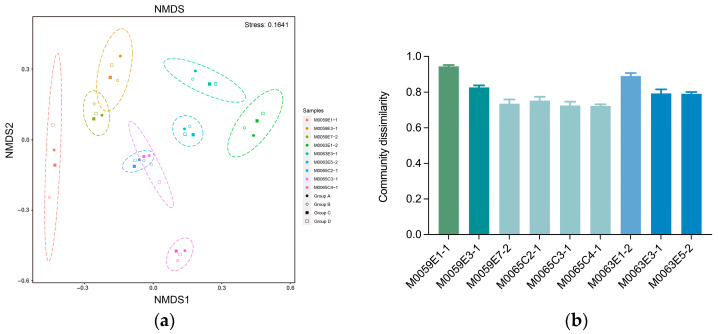
(**a**) A NMDS analysis of a Bray−Curtis dissimilarity matrix. The 95% confidence ellipse was added for each sample. (**b**) Dissimilarities among different groups of grinding beads from the same sample. The line in data plots indicate median value with SD; different colors indicate that there is a significant difference between the samples (*p* < 0.05).

Non-metric multidimensional scaling analysis showed that inter-sample variation was larger than that among the different groups of grinding beads (Figure 4a). Community dissimilarities among different groups of grinding beads for the same sample were calculated using the Bray–Curtis matrix (Figure 4b). Dramatic differences were observed among samples. Community dissimilarity caused by the grinding beads was significantly positively correlated with the cell abundance and water content but negatively correlated with the depth (Spearman, *p* = 0.036, *p* = 3.3 × 10^−5^, and *p* = 0.036, respectively).

**Figure 5:**
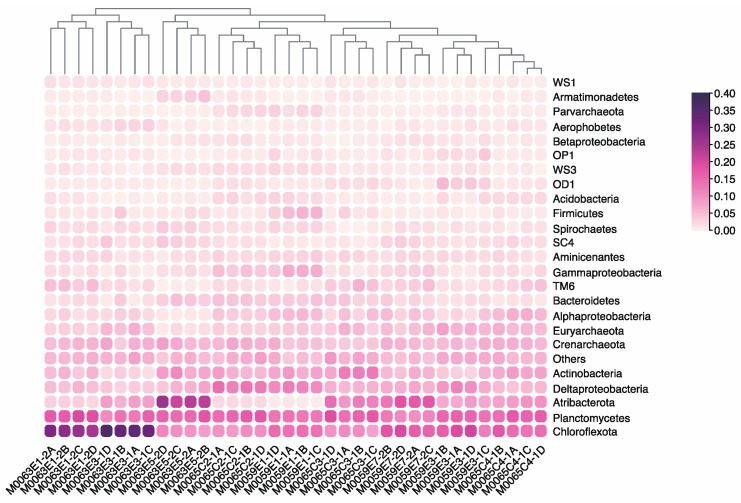
Comparison of the phylogenetic composition at phylum level. The relative abundance is visualized as the changes of colors.

**Figure 6:**
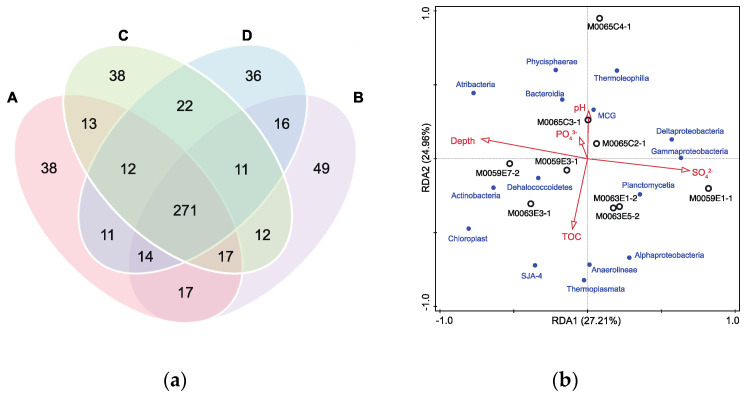
(**a**) Venn diagram displaying the number of unique and shared genus for different combinations of grinding beads. (**b**) Redundancy analysis of ASV abundance constrained by the depth, TOC content (%), pH, concentrations of SO_4_^2−^ (mM) and PO_4_^3−^ (mM). Black labels indicate samples from different boreholes, blue labels indicate dominant taxa at the class level.

The phyla Chloroflexota, Planctomycetes, Proteobacteria, Atribacteria (candidate phylum OP9 and JS1), and Actinobacteria were the most abundant members of the Baltic Sea sediment microbial community, comprising 59.88–74.88% of the sequences. Some rare (relative abundance < 5%) but widespread clades such as Aminicenantes, candidate SC4, and OD1 were detected in this study (Figure 5). Although there was no significant variation in the microbial communities recovered with the four groups of grinding beads from the phylum to the family level, a genus level comparison revealed many unique taxa among the different groups (Figure 6a). Group B obtained more unique genera than the other groups of beads. Generally, the unique genera were rare taxa, except for two unique genera in group B belonging to *Stenotrophomonas* (Gammaproteobacteria) and *Leptotrichia* (Fusobacteria) with contributions of >1% in M0065C3-1. Several genera (*Streptococcus*, *Veillonella*, *Tepidibacter*, and *Ilumatobacter*) affiliated with Firmicutes or Actinobacteria were observed for at least two of the groups of beads but were absent with group D. Overall, group B recovered the most diverse community among the four groups of beads. Group D was likely not suitable for studying the microbial diversity of the Baltic Sea sub-seafloor sediment.

Redundancy analysis was used to study the relationship between the microbial community and parameters (Figure 6b). Among the measured factors, only depth below the seafloor could significantly explain the distribution patterns of the microbial communities inhabiting the sub-seafloor sediment of the Baltic Sea (*F* = 1.76, *p* = 0.023). The relative abundances of Atribacteria and the miscellaneous Crenarchaeotic group (MCG) were positively correlated with depth (*p* < 0.05). By contrast, the percentage of Deltaproteobacteria decreased with depth at both M0059E and M0063E. The opposite vertical distribution pattern was observed for *Chloroplast* at M0059E and M0065C.

## 4. Discussion

The addition of a grinding step with beads increases DNA extraction from sediments [11,27,44]. The beads can break up the clay structure and large organic particles, which enables more efficient penetration of lysis reagents and release of DNA from aggregates and organic debris [30]. Compared with other methods of mechanical disruption of cells (such as sonication), the lysis process with beads is simpler and less expensive. Consequently, grinding with beads is widely used in DNA extraction for diverse environmental samples [26,29,31]. The grinding beads supplied with the Fast DNA^®^ SPIN Kit for soil (1.2 g of 1.4-mm ceramic beads, 0.1-mm silica beads, and one 4-mm glass bead) are designed to extract DNA from a wide range of sources, including soil, marine sediment, feces, sand, and sludge [26,45,46,47]. However, the diameter and material of the grinding beads needs to be optimized for specific sources, such as the sub-seafloor sediment from the Baltic Sea used in the present study. The Baltic Sea has served as a depositional sink throughout at least the last glacial cycle, and its sediment is a unique high-resolution archive of paleoenvironmental history [48]. Extraction of DNA from this sediment is challenging because of the high ancient organic matter content and high biosiliceous clay composition, which can adsorb cells and DNA and affect the recovery [49]. Consequently, beads with small diameters (0.1 mm and 0.5 mm) and low-densities (glass) were selected in this study for DNA extraction from sub-seafloor sediment of the Baltic Sea.

The nine samples used in this study varied in biomass, TOC content, salinity, depth, and other parameters, which led to distinct DNA yields with the four groups of grinding beads (Figure 3a). Unexpectedly, significant differences were observed in the DNA yields obtained with the different groups of grinding beads for one sample that had a high cell quantity and two samples that had low cell quantities. The group A and C beads performed well for low biomass samples, which indicated that cell desorption might play an important role in increasing the DNA yield because these two groups contained more beads with large diameters [50]. When the sample contained large quantities of cells, mixtures of 0.1-mm and 0.5-mm glass beads could both sufficiently desorb cells from clay or large organic particles and break cells with tough cell walls [11,51]. There was a positive correlation between the DNA yield and microbial abundance (Figure 3b) [27,49,52]. However, the good fit between the biomass and DNA yield with the group B beads suggested that the mixture of 0.1-mm and 0.5-mm glass beads was the best choice for DNA extraction from sediments with a high proportion of fine clay minerals. By contrast, the significantly lower DNA yields obtained with group D beads indicated that beads that were too similar in size to the clay-rich sediments might cause insufficient homogenization and desorption. The grinding beads did not appear to affect the DNA purity. Positive relationships between DNA purity and the sulfate concentration suggested that the composition of organic matter in the sediments could affect the residual contamination in the extracted DNA because the sulfate concentration is an indicator of the proportion of recalcitrant organic matter in deep sea sediments [53].

Prokaryotic 16S rRNA gene sequencing results revealed that grinding beads did not affect the richness and evenness of microbial communities in the sub-seafloor sediments from the Baltic Sea. However, among the nine samples, a dramatic difference was observed for the intra sample average community dissimilarity for the four groups of grinding beads (Figure 4b), and this was positively correlated with the microbial abundance (Spearman, *p* = 0.036). This result suggests that grinding beads can influence the beta diversity of microbial communities, and the degree of this effect increases as the biomass content increases. Furthermore, different groups of grinding beads produced many unique clades at the genus level (Figure 6a). The genus *Stenotrophomonas* can efficiently colonize plants, humans, and marine sediments, partly because of the biosynthesis of variable outer membrane lipopolysaccharides (LPS) [54], which can protect the cell structure and absorb DNA [55,56]. The presence of *Stenotrophomonas* and high yields of DNA with group B suggested that the mixture of 0.1-mm and 0.5-mm glass beads could efficiently crush LPS and improve the DNA recovery. Similarly, another unique genus observed with group B (*Leptotrichia*) can also produce diverse LPS [57]. Previous research has indicated that grinding with beads can increase the extraction of DNA from Gram-positive bacteria [58]. The absence of several genera belonging to Firmicutes and Actinobacteria in the DNA obtained with the group D beads suggests that small grinding beads (ø = 0.1 mm) might not break the tougher cell walls of certain Gram-positive strains.

Previous studies have found that Chloroflexota, Planctomycetes, Atribacteria, and Crenarchaeota (MCG) are common in sub-seafloor ecosystems [59,60,61]. In the Baltic Sea, the above clades were also widespread with high relative abundances. The proportions of Atribacteria and MCG greatly increased with depth, which indicated that they adapted to the environment deep under the seafloor. Moreover, Deltaproteobacteria, which are typical heterotrophic anaerobes and sulfate-reducing bacteria [62], were abundant in all samples. An unexpectedly high percentage of green algae belonging to Cyanobacteria were detected in the sediment meters under the seafloor. Growth of Cyanobacteria is generally considered to depend on photosynthesis, but increasing evidence in recent years has found large numbers of Cyanobacteria surviving in dark environments [63,64,65,66]. The green algae found in this study might have been buried by rapidly deposited particles in the Baltic Sea [66] and survived by switching to heterotrophic metabolism [63]. A redundancy analysis revealed that only depth was significantly correlated with the microbial community (Figure 6b). In addition to typical limiting factors (e.g., TOC content, sulfate, ammonium, phosphate, and salinity), some unknown parameters probably play key roles in controlling the distribution patterns of prokaryotic communities in sub-seafloor ecosystems.

## 5. Conclusions

In conclusion, this study demonstrates that the composition of grinding beads can influence the quality of DNA extracted from organic-matter- and biosiliceous-clay-rich samples. In this study, these types of samples were represented by sub-seafloor sediment from the Baltic Sea. A mixture of 0.5-mm and 0.1-mm grinding beads produced high DNA yields and recovered more unique taxa, and may be more suitable than other beads for extraction of DNA from the samples used in this study. This advantage might be magnified in samples with high biomass. Extraction with only small beads might lead to underestimation for certain Gram-positive strains. Overall, the findings of this study provide an assessment of grinding beads with different physicochemical characteristics for DNA extraction from sub-seafloor sediments and an overview of the diversity of microorganisms in the deep biosphere of the Baltic Sea.

## Data Availability

The raw sequences of 16S rRNA gene were deposited in the NCBI GenBank database with the accession number PRJNA897722.

## Supplementary Materials

The following supporting information can be downloaded at: https://www.mdpi.com/article/10.3390/microorganisms10122505/s1, Figure S1: The main physicochemical parameters of M0059E, M0065C, and M0063E with changes in the sediment depth [33,43]; Figure S2: A scheme of the DNA extraction process; Figure S3: Purity of DNA recovered from sediment samples obtained at different boreholes; Figure S4: Comparison of microbial community diversity between boreholes and samples. Table S1: Microbial abundance, physical, and chemical properties of sediment samples; Table S2: Read numbers of 16S rRNA genes of microbial communities obtained using different groups of beads for lysis extraction; Table S3: Alpha diversity indices based on DADA2.
