# Supplementary material for: Grinding Beads Influence Microbial DNA Extraction from Organic-Rich Sub-Seafloor Sediment
Source: Microorganisms. 2022 Dec 18;10(12):2505. doi: 10.3390/microorganisms10122505 (PMC9784657; doi:10.3390/microorganisms10122505)
Supplement: Supplementary file 1 [file microorganisms-10-02505-s001.zip › microorganisms-2047706-supplementary.pdf]

Supplementary Materials for

Grinding Beads Influence Microbial DNA Extraction from Organic-Rich Sub-Seafloor Sediment

Jingjing Niu [1,†], Hong Chen [1,†], Lanlan Cai [2], Maoqiu He [3], Rui Zhang [4] and Long Wang [1,5,*]

[1] State Key Laboratory of Marine Environmental Science, Fujian Key Laboratory of Marine Carbon Sequestration, College of Ocean and Earth Sciences, Xiamen University, Xiamen 361102, China

[2] Department of Ocean Science, The Hong Kong University of Science and Technology, Hong Kong, China

[3] Fishery College, Zhejiang Ocean University, Zhoushan 316022, China

[4] Institute for Advanced Study, Shenzhen University, Shenzhen 518061, China

[5] Southern Marine Science and Engineering Guangdong Laboratory (Zhuhai), Zhuhai 519080, China

[*] Correspondence: kodragon@xmu.edu.cn

† These authors contributed equally to this work.

This file includes:

Figures S1–S4

Tables S1–S3

Supplementary Materials

Table S1. Microbial abundance, physical, and chemical properties of sediment samples.

Sample1	Depth (mbsf)	Prokaryotic Abundance (cells/cm³)	Water Content, Wet Mass (%)	Density, Wet Bulk (g/cm³)	Grain Density (g/cm³)	Fractional Porosity	Void Ratio	TOC (wt%)	pH	Salinity	NH_4^+ (mM)	SO_4^{2-} (mM)	PO_4^{3-} (mM)
M0059E1-1	0.83	9.86×10^8	0.824	1.140	2.440	0.918	11.195	5.445	7.70	26.96	5.90	0.07	1.37
M0059E3-1	7.21	1.16×10^9	0.762	1.176	2.245	0.875	7.032	7.400	7.00	31.81	19.47	0.03	1.81
M0059E7-2	21.82	8.73×10^8	0.645	1.287	2.413	0.811	4.284	5.445	7.72	32.15	36.99	0.03	1.70
M0065C2-1	2.84	6.21×10^8	0.651	1.298	2.586	0.825	4.708	/	7.73	16.40	4.13	0.03	0.44
M0065C3-1	6.47	6.89×10^8	0.629	1.319	2.579	0.810	4.277	4.119	7.84	15.80	4.32	0.01	0.34
M0065C4-1	10.12	2.67×10^8	0.572	1.436	3.105	0.802	4.050	2.832	7.45	13.90	3.30	0.1	0.09
M0063E1-2	2.43	9.66×10^8	/	/	/	/	/	/	7.51	11.80	2.13	0.04	0.32
M0063E3-1	4.30	7.57×10^9	0.690	1.272	2.749	0.856	5.967	/	7.86	13.00	3.90	0.12	0.89
M0063E5-2	9.50	4.60×10^8	0.698	1.251	2.561	0.852	5.767	1.849	7.87	14.50	4.99	0.05	0.90

Abbreviation: mbsf, meters below seafloor. Data from https://doi.org/10.1594/PANGAEA.838369 (accessed on 6 November 2022).

Table S2. Read numbers of 16S rRNA genes of microbial communities obtained using different groups of beads for lysis extraction.

Sample	Raw Sequences	Sample	Raw Sequences	Sample	Raw Sequences
M0059E1-1A	45155	M0065C2-1A	134183	M0063E1-2A	48628
M0059E1-1B	80500	M0065C2-1B	121439	M0063E1-2B	50220
M0059E1-1C	48829	M0065C2-1C	111536	M0063E1-2C	75283
M0059E1-1D	52758	M0065C2-1D	134557	M0063E1-2D	79679
M0059E3-1A	46308	M0065C3-1A	55763	M0063E3-1A	45833
M0059E3-1B	48596	M0065C3-1B	52485	M0063E3-1B	41339
M0059E3-1C	41698	M0065C3-1C	93882	M0063E3-1C	115125
M0059E3-1D	85775	M0065C3-1D	40738	M0063E3-1D	104255
M0059E7-2A	64892	M0065C4-1A	82807	M0063E5-2A	50737
M0059E7-2B	44699	M0065C4-1B	52408	M0063E5-2B	79164
M0059E7-2C	72565	M0065C4-1C	42427	M0063E5-2C	84265
M0059E7-2D	79656	M0065C4-1D	45439	M0063E5-2D	81295

Table S3. Alpha diversity indices based on DADA2.

Sample	Chao–1 Index	Shannon Index
M0059E1-1A	360.10	5.550
M0059E1-1B	673.00	6.183
M0059E1-1C	415.10	5.684
M0059E1-1D	461.50	5.760
M0059E3-1A	263.40	5.290
M0059E3-1B	271.80	5.281
M0059E3-1C	228.10	5.104
M0059E3-1D	401.90	5.642
M0059E7-2A	297.20	5.333
M0059E7-2B	219.30	5.077
M0059E7-2C	376.00	5.526
M0059E7-2D	314.10	5.346
M0065C2-1A	616.10	5.842
M0065C2-1B	594.50	5.923
M0065C2-1C	545.60	5.788
M0065C2-1D	675.60	6.035
M0065C3-1A	293.00	5.190
M0065C3-1B	276.20	5.174
M0065C3-1C	415.80	5.538
M0065C3-1D	198.20	4.902
M0065C4-1A	330.10	5.347
M0065C4-1B	238.50	5.023
M0065C4-1C	206.10	4.988
M0065C4-1D	190.20	4.835

Table S3. Continued.

Sample	Chao−1 Index	Shannon Index
M0063E1-2A	405.10	5.687
M0063E1-2B	403.60	5.726
M0063E1-2C	545.70	6.013
M0063E1-2D	560.30	6.042
M0063E3-1A	269.10	5.227
M0063E3-1B	249.90	5.214
M0063E3-1C	567.00	5.910
M0063E3-1D	557.90	5.906
M0063E5-2A	341.10	5.544
M0063E5-2B	469.20	5.813
M0063E5-2C	478.80	5.821
M0063E5-2D	467.60	5.756
M0063E1-2A	405.10	5.687

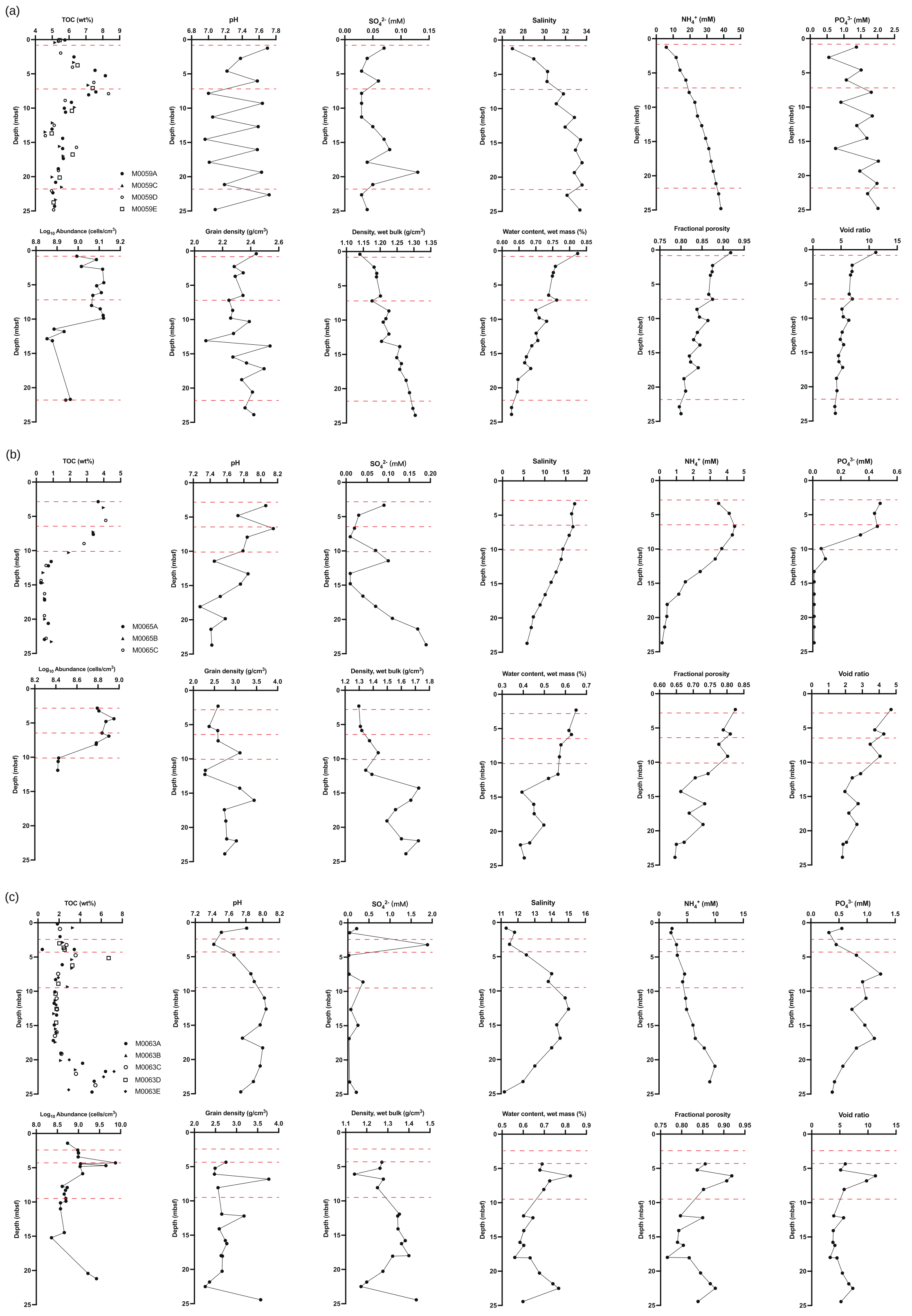

Figure S1. The main physicochemical parameters of (**a**) M0059E, (**b**) M0065C, and (**c**) M0063E with changes in the sediment depth. The red dotted line in the plot indicates the deposition depth of the sample used in this study. The physical and chemical parameters of sediment were taken from the IODP Expedition 347 report [1]. The data for prokaryotic abundance were taken from research on viruses in Baltic Sea sub-seafloor sediments [2].

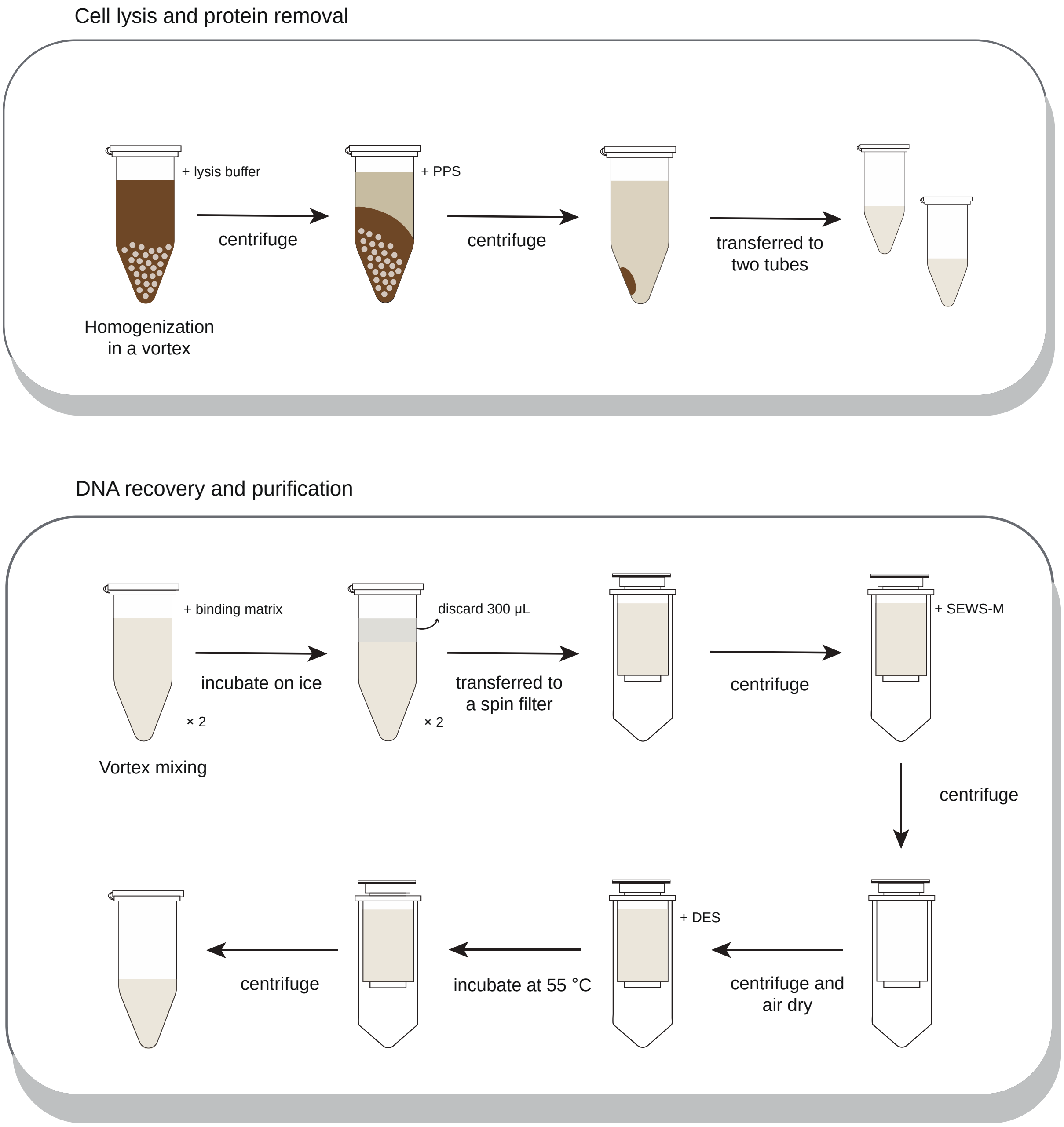

Figure S2. A scheme of the DNA extraction process.

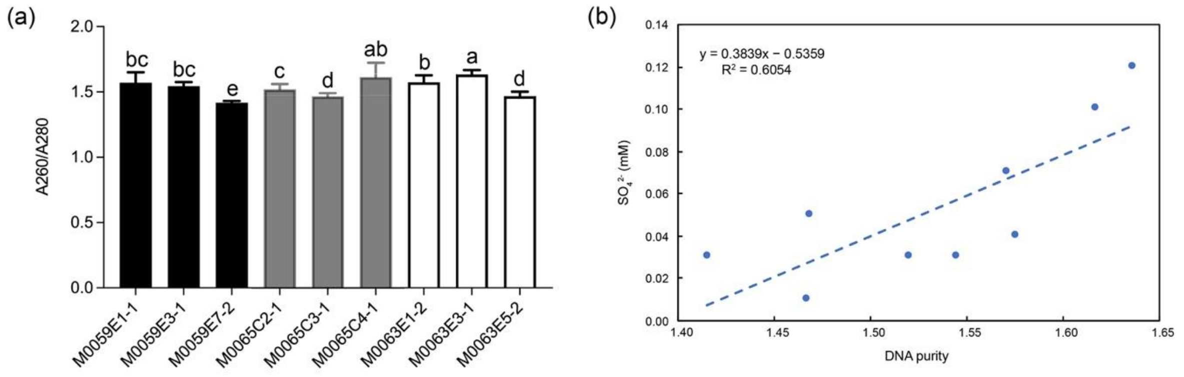

Figure S3. Purity of DNA recovered from sediment samples obtained at different boreholes. (**a**) The lines indicate the median value with the standard deviation. Black, grey and white bars represent the samples from M0059E, M0065C, and M0063E, respectively. The letters a–d indicate that there are significant differences between the samples (p < 0.05). (**b**) Relationship between the SO_4^{2-} concentration and DNA purity (p < 0.05 by linear regression).

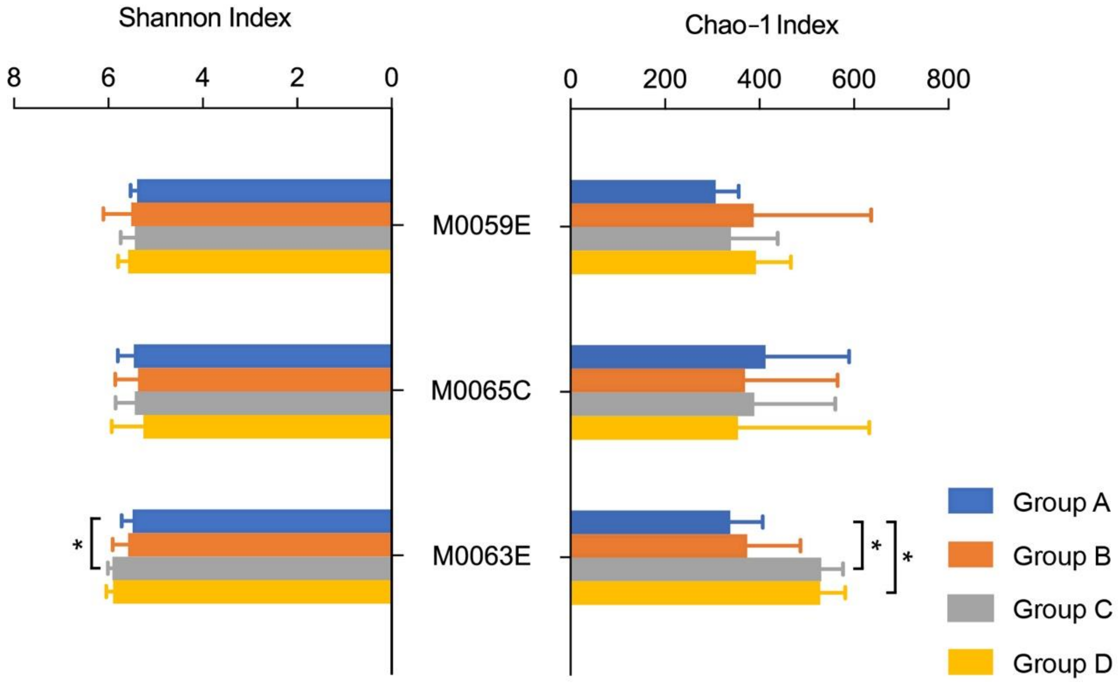

Figure S4. Comparison of microbial community diversity between boreholes and samples. The line indicates the median value with the standard deviation. Asterisks denote significance at $p < 0.05$.

References

1. Andrén, T.; Jørgensen, B.B.; Cotterill, C.; Green, S.; party, the I. expedition 347 scientific IODP Expedition 347: Baltic Sea Basin Paleoenvironment and Biosphere. *Sci Drill* 2015, *20*, 1–12, doi:10.5194/sd-20-1-2015.

2. Cai, L.; Jørgensen, B.B.; Suttle, C.A.; He, M.; Cragg, B.A.; Jiao, N.; Zhang, R. Active and Diverse Viruses Persist in the Deep Sub-Seafloor Sediments over Thousands of Years. *Isme J* 2019, *13*, 1857–1864, doi:10.1038/s41396-019-0397-9.
